# Short-Term Effects of Traditional Greek Meals: Lentils with Lupins, Trahana with Tomato Sauce and Halva with Currants and Dried Figs on Postprandial Glycemic Responses—A Randomized Clinical Trial in Healthy Humans

**DOI:** 10.3390/ijerph191811502

**Published:** 2022-09-13

**Authors:** Emilia Papakonstantinou, Konstantinos Galanopoulos, Anastasia E. Kapetanakou, Maria Gkerekou, Panagiotis N. Skandamis

**Affiliations:** 1 Laboratory of Dietetics and Quality of Life, Department of Food Science and Human Nutrition, School of Food and Nutritional Sciences, Agricultural University of Athens, 75 Iera Odos, 18855 Athens, Greece; 2 Laboratory of Food Quality Control and Hygiene, Department of Food Science and Human Nutrition, School of Food Science and Human Nutrition, 75 Iera Odos, 18855 Athens, Greece

**Keywords:** glycemic index, glycemic response, Lentils, Trahana, Halva, glycemic load, blood glucose

## Abstract

Low glycemic index (GI) diets have been associated with decreased chronic disease risk. In a randomized, cross-over study we investigated the GI and glycemic response to three traditional Greek mixed meals: Lentils, Trahana, and Halva. Twelve healthy, fasting individuals received isoglucidic test meals (25 g available carbohydrate) and 25 g glucose reference, in random order. GI was calculated and capillary blood glucose (BG) samples were collected at 0–120 min after meal consumption. Subjective appetite ratings were assessed. All three tested meals provided low GI values. Lentils GI was 27 ± 5, Trahana GI was 42 ± 6, and Halva GI was 52 ± 7 on glucose scale. Peak BG values were lowest for Lentils, followed by Trahana and then by Halva (*p* for all <0.05). Compared to the reference food, BG concentrations were significantly lower for all meals at all time-points (*p* for all <0.05). Lentils provided lower glucose concentrations at 30 and 45 min compared to Trahana (*p* for all <0.05) and at 30, 45, and 60 min compared to Halva (*p* for all <0.05). BG concentrations did not differ between Trahana and Halva at all time points. No differences were observed for fasting BG, time to peak rise for BG, and subjective appetite ratings. In conclusion, all three mixed meals attenuated postprandial glycemic response in comparison to glucose, which may offer advantages to glycemic control.

## 1. Introduction

Pulses, otherwise known as legumes, and cereals and their products, are the principal components of the Mediterranean diet. Consumption of starch containing foods increases blood glucose concentrations. The extent of the rise depends on many factors including the amount of total carbohydrates and the type of carbohydrate consumed, and other components, including content of dietary fiber, protein, fat, meal and food preparation methods, cooking practices, addition (co-ingestion) of fruits and vegetables, phenolic compounds, etc. High postprandial glycemia has been associated with chronic metabolic diseases such as type 2 diabetes [1,2], cardiovascular diseases [3], greater oxidative stress [4], and increased risk of mortality in people with [5] or without diabetes [6]. The glycemic index (GI) is a tool that classifies the carbohydrate containing foods according to time integrated effects on postprandial glycemia [7,8]. The principle is that the slower the rate of carbohydrate absorption is, the lower the rise of blood glucose level and the lower the GI value will be. GI is defined as the incremental area under the blood glucose curve (iAUC) elicited by 25 or 50 g available carbohydrate portion of a food expressed as a percentage of that after 25 or 50 g carbohydrate from a reference food (typically D-glucose) taken by the same subject [8]. A GI value of ≥70 is considered high, a GI value 56–69 is considered moderate, and a GI value ≤55 is low on the glucose scale [8]. The glycemic load (GL) is the product of GI and the total available carbohydrate content in a given amount of food [1]. It has been shown that the GL is also a good predictor of the level of postprandial glycemia associated with a particular food [9].

Legumes, pasta, and cereal-based meals are fundamental foods of the Mediterranean diet. Dietary interventions for diabetes prevention have frequently focused on foods with a low GI [10], among which pulses stand out not only for producing ameliorated glycemic responses [11], but also for other positive nutritional attributes including high amounts of dietary fiber, micronutrients, phytochemicals, low amounts of fat, and slowly digestible carbohydrates [12,13]. Among the bioactive compounds contained in legumes, some molecules influence glucose metabolism, due to inhibition of enzymes involved in carbohydrate digestion (α-amylase and α-glucosidase), suppression of glucose absorption in the intestine, and stimulation of insulin secretion from pancreatic β-cells [14]. Legumes, particularly lentils, are classified as a low GI food, with GI values ranging from 18 to 53 and GL values ranging from low (i.e., 3) to medium (i.e., lentil soup GL = 12) [11]. The GI of pasta, particularly Tarhana, ranges from low (GI = 20; GL = 4) [15] to high (GI 86–109) [16] values depending on the flour, cereal, and in tarhana’s case, the type of yogurt, used. Halva (halwa), a traditional Greek dessert, is reported to be a low GI (GI = 52–55) and medium GL (GL = 18–19) food [17]. Many factors, such as inclusion of soluble dietary fiber (i.e., beta-glucans), resistant starch and amylose, inclusion of non-cereal ingredients (i.e., fruit fiber, legume-based flours), and flour water content may influence the glycemic response [1,18,19,20,21,22]. One study showed that wheat flour enrichment with vegetables (20%) and beans (25%) powder significantly reduced postprandial glucose of the tested products [23]. Similarly, another study demonstrated that incorporation of chickpea flour (25%) into spaghetti resulted in a significantly lower GI (58.9) compared to traditional spaghetti (72.8) [24]. One study reported that cereal with milk, along with fruits and nuts at breakfast, had a lower and stable glycemic response among healthy subjects [25]. Thus, if a low GI food (i.e., vegetables, fruits, almonds) is to be included in a high GI carbohydrate cereal meal by equi-carbohydrate (approximately iso-energetic) substitution of cereal meal, one might expect greater reductions in glycemic response as the co-ingestion of protein, fat, and fiber, along with carbohydrates reportedly delaying gastric emptying [26].

Pulses, cereals, and pasta are rarely consumed in isolation, so it is best to evaluate their postprandial glycemia lowering effect in the context of mixed meals. It has been found that the same weight of carbohydrate in different foods can produce different postprandial glucose responses. International Tables are available that compare the GI of many different foods [11,27], the implication being that this approach will be helpful in planning meals for individuals with and without diabetes. 

This study was designed to evaluate the short-term effects of three traditional Greek mixed meals, made with modified recipes containing vegetables and/or dried fruits potentially, resulting in higher dietary fiber content compared to meals made with the original recipes: (a) Lentils with lupins for legumes category (Legume), (b) Trahana with tomato sauce for pasta category (Trahana), and (c) semolina Halva with currants and dried figs for dessert category containing fruits and cereals (Halva), all in dehydrated form and consumed after reconstitution with water, on postprandial blood glucose responses. 

## 2. Materials and Methods

### 2.1. Participants

Twelve healthy volunteers (7 males and 5 females), aged 18–65 years, participated in this study. For volunteers’ recruitment a variety of methods were used, including notices posted around the university campus, advertisements online, and flyers. For sample size calculation the *t* distribution was used, with the assumption of an average CV of within-individual variation of incremental area under the curve (iAUC) values of 25%. n 10 participants have 80% power to detect a 33% difference in iAUC with two-tailed *p* < 0.05. 

Before study participation, all volunteers underwent an initial screening that included anthropometry (body weight and height), body composition analysis via bioimpedance analysis (InBody 230, Biospace, Cerritos, CA, USA), and fasting blood glucose via finger prick (calibrated MediSmart^®^ Ruby glucose meter, Lilly-Pharmaserv, SA, Athens, Greece). Participants completed a general health questionnaire. All participants were non-smokers, had a healthy body mass index (18.5–24.9 kg/m^2^), had a normal BP (systolic BP <120 mmHg and diastolic BP <80 mmHg), normal fasting blood glucose levels (<100 mg/dL), and no reported health conditions, including diabetes mellitus, cardiovascular or coronary heart conditions, liver or kidney diseases, severe depression, or gastrointestinal disorders, nor taking medications known to affect glycemia (glucocorticoids, metformin, thyroid hormones, thiazide diuretics), and were not allergic to the test foods. Additionally, women were not pregnant/lactating, nor were they diagnosed with polycystic ovary syndrome. All 12 participants completed all treatments and were included for analysis.

The study was conducted at the Laboratory of Dietetics and Quality of Life, Agricultural University of Athens, Greece. All subjects gave their informed consent for inclusion before participating in the study. The study was conducted in accordance with the Declaration of Helsinki, and the protocol was approved by the Bioethics Committee of the Agricultural University of Athens (EIDE Reference Number 40). This trial was registered at Clinicaltrials.gov (NCT04831268).

### 2.2. Study Design

The glycemic indexes (GIs) of three dehydrated and reconstituted with water typical Greek mixed meals were evaluated. The meal GI was determined following the ISO 26642:2010 International Organization for Standardization [7] methods and procedures. The study consisted of six dietary treatments in a randomized, open-label, cross-over design: three glucose reference drinks as reference food, and a Legume meal, a Trahana (or tarhana) meal, and a semolina Halva (or halwa) meal. Eligible participants were studied on six separate days over a period of 3–9 weeks with an interval of no less than 40 h and more than 2 weeks between tests. An online computer software (Social Psychology Network, Middletown, CT, USA) was used for the simple randomization of the sequence of the test foods (http://www.randomizer.org; accessed on 1 March 2021) [28]. Responsible for the randomization of volunteers to the intervention days examining the test foods was a researcher not involved in the collection and analysis of the scientific data. Volunteers arrived at the Lab of Dietetics and Quality of Life around 8:45–9:00 a.m. after an overnight fast of more than 10 h. Volunteers were asked to keep stable dietary and exercise/activity habits throughout the period of study participation, and to refrain from alcohol on the previous evening, and from vigorous exercise on the morning of the dietary intervention. Participants were allowed to consume only the provided foods throughout the test sessions. If any volunteer reported not feeling well or had not complied with the preceding test conditions, the session was interrupted and rescheduled for another day. Participants’ body weight was measured at every test occasion. Test foods had to be consumed at a comfortable pace within 12–15 min and the glucose beverage within 10 min. Metabolic blood parameters were measured for 2 h post-meal. Participants received in random order the reference food (D-glucose), tested three times (i.e., 1st, 4th, and 6th visit), and the three test meals: Legume, Trahana, and Halva, tested once, in random sequence and at different weeks as recommended by the GI protocol [7]. All the test foods and the reference foods contained 25 g available carbohydrates (378 g Legume, 284 kcal, diluted in 288 mL water; 155 g Trahana, 270 kcal, diluted in 118 mL water; 84 g Halva, 308 kcal diluted in 38 mL water; all served in equal pots). Test meals were given as 25 g available carbohydrates because the portion sizes of the mixed meals were unreasonably large to consume, following the ISO 26642:2010 International Organization for Standardization [7].

The mixed meals were designed and created at the Laboratory of Food Quality Control and Hygiene, Department of Food Science and Human Nutrition, Agricultural University of Athens, Greece. The food products were ready to cook meal-pots. Meal-pots consisted of dried discrete ingredients of 85 g each, which after rehydration, instantly created healthy traditional meals within a maximum of 10 min. The reconstitution took place by adding a half or a whole cup (based on the instructions) of water (38 °C). After that, the meal-pot’s ingredients were agitated, the lid was placed back on the top of the pot, there was a wait for 5 or 10 min, and the meal was ready. After consumption, the sugarcane bio-laminated pot was either recycled or dug in the ground as a compost material. 

The available carbohydrates were determined at the final cooked product (ready to eat boiled and dehydrated meals). The test meal nutritional characteristics that were measured included protein content (Kjeldahl AACC 4712), ash content (AOAC 923.03), moisture (AOAC 930.15), available carbohydrates (available carbohydrates that can be absorbed: sugars and digestible starch, neglecting dietary fiber and resistant starch, and total dietary fibers (Megazyme kit-K-ACHDF, Megazyme Ltd., Scotland, UK). The total meal characteristics and macronutrients composition of the dried test foods are shown in Table 1. Trahana is fermented traditional pasta, mixed with sun-dried tomato sauce including zucchini, leek, celery, pepper, onion, and carrot, tomato powder, and spray-dried olive oil. Legume meal was made of lupin (*Lupinus* spp., Fabaceae legume family), seeds, and lentils. It contained spray dried olive oil (60% *w*/*w* of extra virgin olive oil) as an emulsifier agent and mixed with a vegetable mix (zucchini, leek, celery, pepper, onion, spinach, and carrot), tomato powder, dried olive paste, and spray-dried olive oil. Halva was roasted semolina (10 min) mixed with fruits (currants and dried figs), roasted almonds, and spices (cinnamon, carnation, and licorice). The above mixed meals were portioned at 85 g and packed in ecological pots made of bio-laminated sugarcane.

### 2.3. Blood Glucose Concentrations

Two fasting blood samples were obtained on each test food session by finger-prick at 5-min intervals (−5, 0). The average of the glucose concentrations at these two time points was taken to be the baseline (fasting) concentration. Then, participants were served a test meal along with 250 mL water. Additional capillary blood samples were collected at 15, 30, 45, 60, 90, and 120 min post-meal. Each blood glucose time value recorded was the mean of three blood samples taken from the same drop of blood of every volunteer. A blood glucose test record was filled before and during all test sessions that included the volunteer’s initials, identification number, date, body weight, test food consumed, beverage (type and quantity) consumed, time of starting to eat, time it took to consume the test meal, time and composition of last meal, and any unusual activities. During the 2 h test session, volunteers were asked to remain seated quietly. A snack was offered to participants after the last blood sample was obtained, and volunteers were allowed to depart. For data collection procedures standardization, capillary blood glucose measurement was performed at the fingertip (distal phalange of the third finger). Blood glucose was measured using glucose dehydrogenase-FAD test strips (Ruby Blood glucose Test Strips, Lilly-PHARMASELV S.A., Athens, Greece) which show no reactivity to any sugars other than glucose and have better heat resistance and oxygen resistance with calibrated glucometers. 

**Figure 1:**
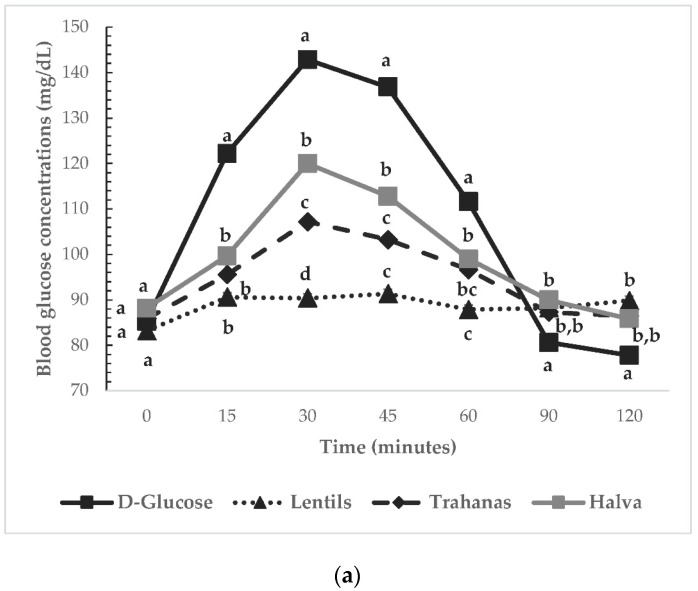

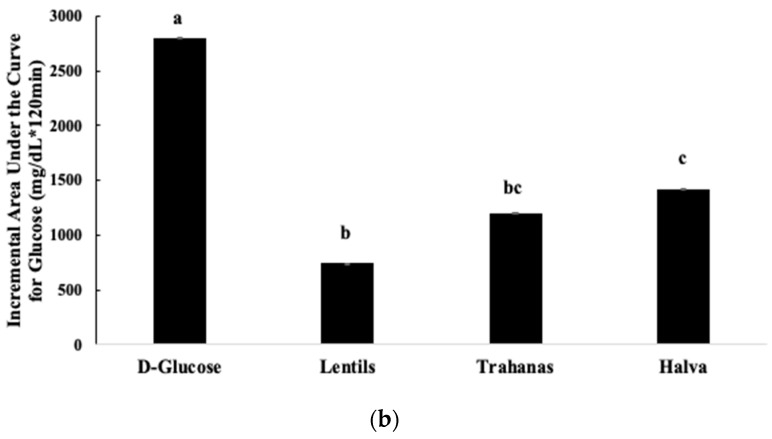
(**a**). Mean blood glucose responses elicited by the reference food (D-glucose 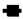), lentils and lupins (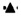), trahanas with tomato sauce (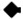), and halva with currants and dried figs (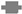) (*n* = 12). Data were compared by post hoc analysis of repeated measures ANOVA. The models included the factors “subject” (id), “sequence” for inter-subject variation, and “period” and “treatments” to account for intra-subject variability. Values marked with different superscript letter are significantly different (*p* < 0.05). (**b**). Incremental area under the glycemic response curve (iAUC) elicited by the reference food (D-glucose), lentils and lupins, trahanas with tomato sauce, and halva with currants and dried figs (*n* = 12). Data are the means ± S.E.M. Post hoc Tukey test, with Bonferroni correction, was conducted to determine test meal differences. Values marked with different superscript letter are significantly different (*p* < 0.05).

We chose to determine the GI of test foods using a calibrated glucometer, as it has been adequately reported that GI results obtained by glucometers are reliable and similar to those attained by enzymatic kits methods [29,30,31]. The repeatability and within laboratory CV were 3.2%. The mean blood glucose values of all volunteers at each time point were plotted as the average blood glucose response curve (Figure 1a). For each sample and participant, iAUC was calculated using the trapezoid rule geometrically, ignoring the area beneath the baseline [7]. GI of every test meal was calculated as the mean of the ratios. For every participant, the ratio between the individual iAUC after consumption of the test meal and the iAUC for the same participant after consumption of the reference food was calculated and expressed as a percentage value. The GI of each test meal was calculated as the average value of the ratios across all participants consuming the test meals. The peak blood glucose (highest recorded glucose value minus baseline) and the peak blood glucose time (time elapsing from the start of a test meal to the highest recorded blood glucose value) were calculated. 

### 2.4. Subjective Appetite 

Subjects rated their hunger, desire to eat, and perceived fullness after eating on 100 mm line visual analogue scales (VAS), ranging from not at all (0 mm) to extremely (100 mm), with, for example, neither hungry (0 mm), full (100 mm), or having desire for food in the middle (50 mm). VAS were given in the form of a booklet, one scale per page [32]. VAS ratings were obtained at times 0, 15, 30, 45, 60, 90, and 120 min post-test meal consumption. 

### 2.5. Dietary Intake

Dietary intake was assessed by 24 h recalls at every visit, and analyzed using the Diet Analysis Plus program, as well as using Hellenic and European Food Composition Databases (http://www.eurofir.org/foodinformation/food-composition-databases-2; accessed on 1 March 2020). The databases were modified to include new foods and recipes. The purpose of collecting dietary intake was to confirm that participants refrained from changing their eating habits until the study was completed. 

### 2.6. Statistical Analysis

Data is presented as mean ± standard error of the mean (SEM), unless otherwise specified. Data distribution was tested by kernel density plots. Analysis of variance (ANOVA) was used to evaluate the differences in baseline continuous variables with normal distribution. Kruskal–Wallis test was used to evaluate skewed continuous data. Pearson’s chi square test was used for categorical variables. Two different researchers entered the data into a spreadsheet. All values were compared to assure accurate transcription. iAUCs were calculated ignoring the area below fasting. The fasting blood glucose value was the mean of the first measurement of blood glucose values at times −5 and 0 min, and this was used for the purposed of AUC calculations. The test meal GI was calculated by expressing each volunteer’s AUC for the test food as a percentage of the same volunteer’s mean AUC for the three D-glucose beverages (reference food). If the calculated values had more than two SD above the mean, they would be excluded. No GI outliers were found. The ISO method requires, for a valid GI measurement, that the mean within-individual coefficient of variation of glycemic responses elicited by repeated tests of oral glucose (termed reference CV) is ≤30% [7]. We calculated reference CV for glucose according to the ISO method; namely the mean, SD, and CV (100 × SD/mean) of the glucose iAUC values elicited by the two repeated tests of 25 g glucose were calculated for each participant and the mean of the resulting values was the reference CV. The blood glucose concentrations at each time, AUC, GI values, subjective appetite, and BP, were subjected to repeated-measures ANOVA examining for the main effects of test food and the food x participant interaction. ANOVA for a 2 × 2 crossover study was conducted for blood glucose concentrations between treatments. In such a 2 × 2 design, one assumes that there are no individual effects because a complete randomization procedure was followed for dietary treatment allocation. The models used included the factors “id of participant”, test food “sequence” for inter-individual variation, “period”, and “test meal” to account for intra-individual variability. The time × test meal interaction was evaluated. Post hoc analysis using the Tukey test with Bonferroni correction was used to evaluate multiple comparisons between the test food interventions. One-way ANOVA was used to evaluate differences between test meals followed by post hoc Tukey test with Bonferroni correction in all other parameters. BP differences were assessed with Paired T-test analysis. Differences in VAS ratings were evaluated using one-way ANOVA and the Friedman’s test. Correlations between GI and test meals’ characteristics were determined with Spearman’s rho coefficient. Means differing by more than the LSD (least significant difference) were statistically significant, two-tailed *p* < 0.05. The GL (g glucose equivalents)/1000 kJ values were calculated by multiplying the amount of available carbohydrate contained in a 1000 kJ portion of that food as well as the amount of available carbohydrate contained in an 85 g meal-pot portion, which was then divided by 100. Data were analyzed using SPSS 20.0 software (SPSS Inc., Chicago, IL, USA).

## 3. Results

### 3.1. Test Meals

All three mixed meals had similar energy content. Trahana was classified as high fiber food as it contained 12.4 g (>6 g) of fiber in 100 g of the product, and a source of protein since 12.3% (>12%) of energy came from protein. Legume was classified as high protein food since 33% (>20%) of energy came from protein, and a high fiber food as it contained 22.1 g (>6 g) of fiber in 100 g of the product. Halva was classified as a high fiber food as it contained 6 g of fiber in 100 g of the product, a salt-free food as it contained 0.009 g (<0.10 g) of salt in 100 g of product, and a low saturated fat food as it contained 0.9 g (<1.5 g) of saturated fat in 100 g product, and high unsaturated fat food given the fact that 92% (>70%) of the fatty acids present in the product derived from unsaturated fat and at the same time unsaturated fat provided 21% (>20%) of the energy of the product. Halva was also a no added sugars food, given the fact that it did not contain any added mono- or -disaccharides or any other food ingredient or additive used for its sweetening properties.

### 3.2. Participants’ Baseline Characteristics 

The participants’ characteristics can be found in Table 2. There were no intermittent missing values or dropouts. 

#### 3.2.1. Glycemic Index (GI) of the Tested Meals 

The results of GI and GL for the three test meals are presented in Table 3. According to the current classification [7,8], all three mixed meals are classified as low GI foods (GI ≤ 55 on glucose scale; Legume: GI = 27; Trahana: GI = 42; Halva: GI = 52). Legume and Trahana were classified as low GL (GL ≤ 10 per 85 g serving; Legume: GL = 1.5; Trahana: GL = 6) and Halva was classified as medium GL (GL: 11–19 g/85 g serving; Halva: GL = 13) food. GL values were calculated for the 85 g serving, as well as for 1000 kJ and not related to portion as previously suggested [18], because in some foods it may be difficult to define the serving size for each item since portion sizes vary markedly among food industries and consumers. Compared to glucose, all three test meals, Legume, Trahana, and Halva, had significantly lower GI and GL values. Legume had significantly lower GI and GL compared to Trahana and Halva. There were no significant differences in GI and GL between Trahana and Halva (Table 3). 

#### 3.2.2. Glycemic Responses to Mixed Meals

The change in postprandial glucose over time (120 min) can be seen in Figure 1a. No significant differences were observed in fasting glucose concentrations between glucose and the test meals (*p* for all >0.05). There was a significant blood glucose × time interaction (*p* < 0.001), a blood glucose × time × test meal interaction (*p* < 0.001), and a time × test meal interaction (*p* < 0.001). There was a main effect of test meal on blood glucose concentrations (*p* < 0.001). Compared to the reference food (D-glucose), lower blood glucose concentrations were observed after the consumption of all test meals at all time points (*p* for all <0.05). Legume provided lower glucose concentrations at 30 and 45 min compared to Trahana (*p* for all <0.05) and at 30, 45, and 60 min compared to Halva (*p* for all <0.05; Figure 1a). Blood glucose concentrations did not significantly differ between Trahana and Halva at all time points (*p* for all >0.05; Figure 1a). There was a significant main effect of meal on peak blood glucose values (*p* < 0.001). Peak glucose values were significantly lower for all test meals compared to the reference food (D-glucose; *p* < 0.001; Table 3). Halva had significantly higher peak glucose value compared to Legume (*p* < 0.001) and Trahana (*p* = 0.020) (Table 3). Legume tended to have lower peak glucose value compared to Trahana (*p* = 0.066) (Table 3).

The iAUC 120 min for blood glucose values calculated for each test meal are shown in Table 3 and Figure 1b. There was a significant main effect of meal on iAUC for blood glucose (*p* < 0.001). The mean within-individual variation of iAUC for blood glucose for the repeated tests of glucose was 14%. The iAUC for blood glucose values calculated for all three meals were significantly lower than those of the reference food (D-glucose; *p* for all <0.001). Legume had a significantly lower iAUC for blood glucose only compared to Halva (*p* < 0.001), without differences with Trahana (*p* = 0.29). No differences were observed for time to peak rise for blood glucose and subjective appetite ratings at all time points between mixed meals and reference food (*p* for all >0.05).

## 4. Discussion

This study aimed to evaluate the postprandial glycemic responses of three traditional Greek mixed meals, commonly consumed carbohydrate sources, using novel recipes, packages, and methods of delivery and consumption. The main findings of this study were that: (a) mixed traditional Greek meals produced different GI/GL, (b) Legume meal produced lower GI/GL, lower peak blood glucose values and lower overall glycemic responses, followed by Trahana, and then by Halva when compared to the reference food (D-glucose). This study demonstrated that a starch-rich food, such as Trahana or Halva when mixed with vegetables and fruits, respectively, in a mixed meal significantly reduced postprandial glycemic responses and peak blood glucose values compared to the reference food (D-glucose). We demonstrated that Legume, Trahana, and Halva mixed meals all lowered postprandial glycemia, which represented a 73%, 57%, and 49% reduction in glycemic response, respectively, compared to the reference food. Moreover, all meals produced lower peak blood glucose values compared to the reference food. It has been proposed that products characterized by lower peak blood glucose values indicate a favorable postprandial glycemic response [33]. 

Lentils (red, green) are classified as a low GI food [27]. Results of the current investigation regarding lentils’ postprandial blood glucose lowering effects are in agreement with a recent review of the literature, concluding that lentil consumption lowers acute blood glucose and insulin responses consistently when compared to starchy control foods, but the mechanism is still unclear [34]. It was discussed that the minimum effective serving of lentils is about 110 g cooked to reduce postprandial blood glucose concentrations by 20% [34]. Reductions in postprandial blood glucose levels were correlated mainly to lentils’ protein (45–57 g) and dietary fiber (22–30 g) content, and only weakly to available carbohydrate content [34]. Both high (>100 g) and low (<100 g) lentil serving sizes had a favorable postprandial blood glucose and insulin lowering effect, making it difficult to identify the optimal lentils’ serving size for beneficial dose response effects [34]. Results of the current investigation are consistent with another study that reported the lowest GI and glycemic responses after consumption of lentil soup with bread roll compared to five other starchy mixed meals [35]. Another study found a 71% reduction in glycemic responses after lentils with butter and tomatoes consumption compared to two wholemeal bread meals [36], similar to the 73% reduction observed in the current investigation. Four studies have reported that the lentils’ GI and glycemic responses lowering effects may be due to their moderate fraction of rapid carbohydrate digestion rate [37] and resistant starch content [38,39]; leading to a release of only 39% of the sugars and oligosaccharides liberated from bread and raising postprandial blood glucose concentrations by only 42% of the bread value in both healthy volunteers and diabetics [40]. Lentils and lupins mixed meal was superior to other mixed meals possibly due to their higher protein and total dietary fiber content. The recorded attenuation of postprandial blood glucose in response to the legume meal is an effect thought to be due to the ability of protein to delay gastric emptying and increase postprandial insulin responses [38]. Further, total dietary fiber has long been suggested to attenuate postprandial blood glucose by increasing the viscosity of intestinal contents [41]. Additionally, it has been suggested that the phenolic compounds (mainly flavonols) in lentils interfere with starch digestion and hydrolysis due to their ability to complex with the amylose faction of starch reducing the *α*-glucosidase activity, the main intestinal enzyme involved in starch digestion, further increasing the resistant starch content [42]. 

Tarhana has produced inconsistent effects on glucose excursions and glycemic responses, with one study showing that it ameliorated the glycemic responses in both healthy subjects and diabetics [15], and another showing that it may produce even higher than D-glucose consumption glycemic responses [16], depending on the flour, type of yogurt, and type of cereal used. An older review discussed that Trahana is one of the most popular fermented milk-cereal products of Greece which is produced during summer mainly from whole fresh ewes’, goats’ milk, or a mixture of them [43]. Acidified (sour) milk or sweet milk or a pulp of vegetables in times of religious fasting periods is used and the final products are called sour or sweet trahana, respectively [43]. Typically, trahana made without milk contains about 71 g/100 g product carbohydrates, protein 12 g, fat 3 g, dietary fiber 1 g, and salt 2 g/100 g dry matter [43]. Trahana made with milk and called ‘sour’, ‘sour with eggs’, and ‘sweet’ contains about 68 g/100 g product carbohydrates, protein 15 g, fat 4 g, dietary fiber 1.5 g, and salt 2 g/100 g dry matter [43]. In the current investigation study, the Trahana with tomato sauce mixed meal produced in our research center differed greatly from the average Trahana studied in other trials as it had significantly lower total carbohydrates content and almost 10 times more dietary fiber, which may explain the 57% reduction in postprandial blood glucose responses reported. The GI of Trahana with tomato sauce of 42 is similar to results obtained and recently reported by our group for three different types of spaghetti No 7 (regular, whole grain, and a novel low carbohydrate—high soluble fiber spaghetti) [44]. The low GI of pasta has also been suggested by others [11,37,45,46]. Several factors have been described as responsible for the low rate of digestibility of pasta compared to rice or bread; one of which being the microstructure formed as a result of the technological process of pasta making causing the encapsulation of starch granules in the gluten matrix [47].

The results of our current investigation regarding halva (halwa) are in agreement with another study examining the GI and GL of two types of Omani halwa (white and black) that reported low GI, moderate GL values for both types (white: GI = 55, GL = 14; black: GI = 52, GL = 13, respectively) [17]. One study reported a 52% lower glycemic response for cereal with milk, along with fruits and nuts breakfast meal when compared to glucose as reference food [25]. Another study reported a flattened glycemic response after the inclusion of 13 g of protein and 10 g of dietary fiber in a cereal-based bread, consisted of 22% dried fruits (figs, apricots, raisins, and prunes), which were both 1.7 fold higher than the amount provided in the present study [48]. These effects primarily could be explained not only by the higher fiber content in the cereal-based bread, but also by the presence of added proteins. Intake of soluble dietary fiber has been negatively correlated with postprandial glucose and insulin responses [49]. These effects may be related to the increased viscosity of the meal bolus in the stomach, reducing the mixing of the food with digestive enzymes and gastric emptying, leading to a delayed digestion of starch and, therefore, delayed absorption of glucose [50]. The results of the current investigation regarding ameliorated postprandial glycemia after consuming high carbohydrate cereal foods along with fruits were in agreement with another study supplementing starch (i.e., white bread) with a mixture of polyphenol and fiber-rich foods (i.e., green tea powder, apple peel, blackberry, blackcurrant, and strawberry freeze-dried powders) [51]. The incorporation of low-GI carbohydrate foods such as lentils and lupins, tarhana, and cereals with fruits and almonds into the diet reduced the postprandial glucose profile, suggesting that the choice of carbohydrate-rich food is important and that the glycemic index approach in dietary planning is useful.

### 4.1. Study Limitations and Advantages

The strength of our study includes the randomized, crossover design where each subject served as his own control. The major limitation of the current investigation is the acute feeding protocol which does not allow the translation of the acute findings to long-term benefits. Another limitation is the small sample size, although the use of twelve volunteers has been validated by many studies. Nonetheless, this sample size lowers the study precision and may lead to exaggerated associations. Moreover, blood collections from the participants would enable measurements of plasma insulin and incretins. In addition, this study was conducted in healthy, normal body weight, and normoglycemic young adults. Our results need to be confirmed in other populations, i.e., middle-aged or elderly people with prediabetes or diabetes with and without obesity.

### 4.2. Practical Applications

To the best of our knowledge, this study determined, for the first time, the GI of three traditional Greek mixed meals made with modified recipes, including vegetables and/or dried fruits, and containing significantly more dietary fiber compared to the same meals prepared with use of original recipes: Lentils and lupins, Trahana with tomato sauce, and Halva with currants and dried figs. Our study followed controlled circumstances for a GI protocol. Based on our results, all three mixed meals, Legume, Trahana, and Halva, produced ameliorated blood glucose responses when compared to the reference food. Moreover, consumption of Legume and Trahana can aid significantly in covering almost 75% and 41%, respectively, of the recommended daily intake of dietary fiber according to WHO, whilst consuming less carbohydrates than other carbohydrate-rich sources such as potatoes or rice in the same portion consumed. Interestingly, Halva, in the proposed modified recipe, can aid in covering 16% of the recommended daily intake of dietary fiber according to WHO, whist being considered salt-free, low saturated fat, high unsaturated fat, and a food without added sugars.

## 5. Conclusions

In conclusion, the results of this study showed that lentils and tarhana are low GI, low GL foods, whereas halva is a low GI, medium GL food. All three mixed meals are considered a suitable dietary alternative for glycemic control as they produced lower postprandial glucose concentrations and lower glucose excursions in young healthy subjects compared to the reference food (D-glucose). Future long-term studies are needed to provide an insight regarding the mechanisms by which different types of legumes, pasta, and cereals with and without fruits and nuts, of varied formulations, lead to ameliorated glycemic responses in different population groups, including obese and non-obese individuals with type 2 diabetes. Improvements in foods’ nutrient density and their impact on chronic disease prevention should be the focus of research centers and the food industry. For example, the addition of vegetables and other healthful ingredients in legumes, pasta products, and deserts, such as halva, is highly encouraged, as these ingredients may aid significantly in covering significant proportion of the recommended daily intake of dietary fiber according to WHO (i.e., in the current investigation 75% for legume, 41% for trahana, and 16% for halva) in the same portion consumed. Consumption of lentils, trahana, and modified halva, with different formulations, higher in dietary fiber and plant proteins, is an important strategy for glycemic control and prevention or dietary treatment of chronic diseases. However, it should be noted that commercial halva should be consumed with caution because of high fat and sugar content.

## Figures and Tables

**Table 1:** Average Nutritional Facts of the mixed meals (per 85 g).

	Trahana	Legume	Halva
**Physicochemical characteristics (at 25 °C)**			
Water Activity (aw)	0.5	0.5	0.4
pH	4.3	4.8	4.4
**Nutritional composition (per 85 g serving)/% Reference Intake (RI)**			
Energy (KJ/ Kcal)	1130/270/RI 14%	1188/284/RI 14%	1289/308/RI 15%
Total fat (g)	2.4/RI 5%	7.2/RI 11%	9.3/RI 14%
Saturated Fat (g)	0.6/RI 3%	1.3/RI 6%	0.8/RI 4%
Polyunsaturated Fat (g)	0.3	0.9	2.6
Monounsaturated Fat (g)	1.5	5.1	5.2
Carbohydrates (g)	48.7/RI 17%	41.7/RI 16%	53.0/RI 18%
Sugars (g)	14.9	6.5	5.1
Dietary fibers (g)	10.5/RI 41%	18.8/RI 75%	5.1/RI 16%
Proteins (g)	8.3/RI 16%	23.5/RI 47%	6.4/RI 13%
Salt (g)	1.0/RI 17%	0.8/RI 13%	0.0/RI 0%
Amount of food providing 25 g available carbohydrates (g) *	155	378	84

* Available carbohydrates and dietary fiber were determined according to the method AOAC 991.43, with the Megazyme assay kit (Bray, Ireland). Abbreviations: Trahana = Greek Trahana (or tarhana) with tomato sauce; Legume = Lentils and lupins with tomato sauce; Halva (halwa) = with dried currants and figs; RI: Reference Intake of an average adult (8400 KJ/2000 Kcal).

**Table 2:** Baseline participants’ characteristics (*n* = 12).

	Total
N	12 (5 men, 7 women)
Age (years)	23.3 ± 3.2
**Body composition (derived by DXA)**	
Weight (kg)	74.4 ± 8.7
BMI (kg/m^2^)	23.7 ± 1.4
Waist circumference (cm)	83.5 ± 8.0
Fat (%)	36.5 ± 10.2
Waist to hip ratio (WHR, cm)	0.9 ± 0.2
**Dietary intake (derived by 24 h recall)**	
Energy (kcal)	1576.5 ± 568.2
Protein (g)	68.9 ± 32.0
Carbohydrates (g)	184.6 ± 60.1
Fat (g)	67.8 ± 29.0

Values are means ± SEM. Abbreviations: BMI: body mass index; DXA: dual energy X-ray absorptiometry.

**Table 3:** Incremental area under the curve (iAUC) for blood glucose, glycemic index (GI), and glycemic load (GL) of three mixed meals, relative to the reference D-glucose (*n* = 12).

Meals	0–120 min iAUC for Blood Glucose (mg·120 min·dL^−1^)	GI (on Glucose Scale)	GL per 85 g Portion	GL (per 1000 kJ)	Peak Glucose (mg/dL)
D-Glucose	2803 ± 108 ^a^	100 ^a^	-	-	61 ± 3 ^a^
Trahana with tomato sauce	1203 ± 186 ^bc^	42 ± 6 ^bc^	6 ± 1 ^bc^	5 ± 3	24 ± 2 ^b^
Lentils and lupins	745 ± 116 ^c^	27 ± 5 ^c^	2 ± 0.2 ^c^	1 ± 1	12 ± 2 ^b^
Semolina halva with dried fruits	1419 ± 165 ^b^	52 ± 7 ^b^	13 ± 2 ^b^	10 ± 3	38 ± 4 ^c^

Mean incremental area under the curve (iAUC), glycemic index (GI), glycemic load (GL), and peak glucose values elicited by the reference food (D-glucose), lentils and lupins, trahanas with tomato sauce, and halva with currants and dried figs. Data are the means ± SEM. Each value represents the mean of twelve participants. For each sample and each study subject, the incremental area under the curve (iAUC) was calculated geometrically, using the trapezoid rule, ignoring the area beneath the baseline. The glycemic index (GI) calculation for each test food sample used the method referred to as the mean of the ratios. For each subject, the ratio between the individual iAUC after consuming the test food sample and the iAUC for the same subject after consuming the reference food was calculated and expressed as a percentage value. Then, the GI of each test food was calculated as the average value of the ratios across all the subjects consuming the test food samples. The glycemic load (GL) (g glucose equivalents)/1000 kJ values were calculated by multiplying the amount of available carbohydrate contained in a 1000 kJ portion of that food as well as the amount of available carbohydrate contained in an 85 g meal-pot portion, which was then divided by 100. Values marked with different superscript letter are significantly different (*p* < 0.05). Means were compared column-wise by using one-way ANOVA for factor “treatment”, period and sequence of treatment, and post hoc Tukey test with Bonferroni correction to account for multiple comparisons between test meals; *p*-values < 0.05 were considered as significant.

## Data Availability

Not applicable.
